# Endoprosthetic Reconstruction for a Displaced Atypical Femoral Fracture in a Cancer Patient with Poor Prognosis

**DOI:** 10.1155/2018/7862516

**Published:** 2018-09-20

**Authors:** Hironari Tamiya, Hiroki Hagizawa, Takaaki Nakai, Yoshinori Imura, Takaaki Tanaka, Kazuya Oshima, Toshikazu Ito, Norifumi Naka, Shigeyuki Kuratsu

**Affiliations:** ^1^Department of Orthopedic Surgery, Bellland General Hospital, 500-3 Higashiyama, Naka-ku, Sakai, 599-8247 Osaka, Japan; ^2^Department of Musculoskeletal Oncology Service, Osaka International Cancer Institute, 3-1-69 Otemae, Chuo-ku, Osaka City, 541-8567 Osaka, Japan; ^3^Department of Surgery, Rinku Genaral Medical Center, 2-23 Rinku Ourai Kita, Izumisano, 598-8577 Osaka, Japan

## Abstract

Zoledronate or denosumab treatment is beneficial for cancer patients with bone metastasis. However, each agent may trigger atypical femoral fractures. Incomplete atypical femoral fractures can be successfully treated with prophylactic intramedullary nailing. On the other hand, intramedullary nailing for displaced atypical femoral fractures occasionally causes problems with regard to bone healing, resulting in long-term treatment. In cancer patients with poor prognosis who experience atypical femoral fractures, improvement in activities of daily living should be the priority. Thus, we performed endoprosthetic reconstruction for a displaced atypical femoral fracture in a breast cancer patient with poor prognosis to enable walking in the early stage after the operation. Two weeks after the operation, she could successfully walk. The postoperative Musculoskeletal Tumor Society score was 47%, and it had improved to almost the preoperative level before injury (50%). In conclusion, endoprosthetic reconstruction for displaced atypical femoral fractures may be a first-line treatment approach to acquire early postoperative walking ability for improving activities of daily living in cancer patients with poor prognosis.

## 1. Introduction

Although bisphosphonates (BPs) are widely used for osteoporosis, zoledronate, which is a BP, is commonly used to reduce the occurrence of skeletal system-related events in metastatic bone disease. In addition, the anti-RANKL monoclonal antibody denosumab is used for bone metastasis. Zoledronate and denosumab are categorized as bone-modifying agents (BMAs) that effectively inhibit bone destruction by osteoclasts activated via metastatic bone tumors [1]. However, adverse effects, such as BP-related osteonecrosis of the jaw, renal dysfunction, and hypocalcemia, have been reported. Moreover, several clinical studies have shown that long-term administration of these agents can cause atypical femoral fractures (AFFs) [2].

AFFs are more challenging to treat than ordinary femoral fractures because of issues with bone healing. Incomplete AFFs should be prophylactically fixed with intramedullary nailing (IMN) because of the low rate of spontaneous bone healing [3]. For displaced AFFs, IMN is also considered as a standard approach; however, a previous report mentioned that the revision rate associated with delayed union was 46% [4]. Once delayed union/nonunion occurs, nonweight bearing is required for a long time. Furthermore, implant breakage can occur if the fracture site is overloaded under excessive force. In such cases, a longer time will be required for the treatment of displaced AFFs.

Among cancer patients, life expectancy is limited in some patients, and improvement in activities of daily living (ADL)/quality of life (QOL) is a priority in these cancer patients. If delayed union/nonunion occurs, IMN could fail because of insufficient fixation for displaced AFFs in cancer patients. Endoprosthetic reconstruction is commonly used for primary malignant tumors, and there is no concern about bone healing problems, although this procedure is more invasive and costly. We performed endoprosthetic reconstruction for a displaced AFF in a breast cancer patient with a poor prognosis, and the patient could successfully walk in the early stage after the operation. We believe that endoprosthetic reconstruction may enable early postoperative ambulation and improve ADL/QOL in cancer patients with poor prognosis.

## 2. Case Report

A 48-year-old woman with breast cancer underwent mastectomy (histology: invasive ductal carcinoma, histology grade 2; estrogen receptor: positive; progesterone receptor: positive; HER2: positive; Ki67: 10%, n^+^[27/28]) at the department of surgery in a previous hospital. Subsequently, she underwent chemotherapy with paclitaxel and doxifluridine and hormonal therapy with tamoxifen. Six years after surgery, bone metastasis was noted in the vertebra, and she was treated with a combination of radiotherapy and chemotherapy with trastuzumab. However, the metastatic disease progressed. Liver metastasis was also noted at 57 years of age, and the treatment was switched to capecitabine plus lapatinib, which was shortly discontinued because of adverse effects. Disease progression continued, although fulvestrant was also added. Eventually, she underwent chemotherapy with trastuzumab emtansine (T-DM1).

**Figure 1:**
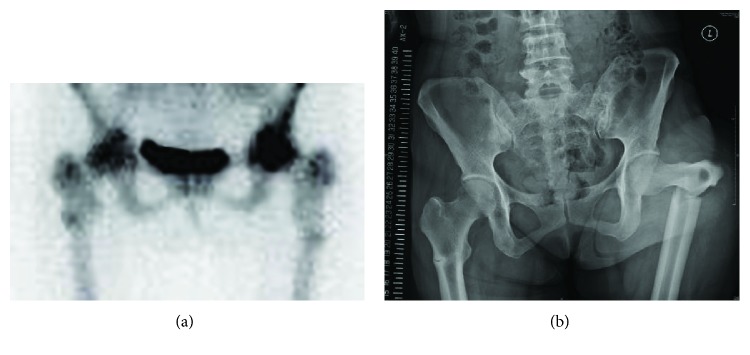
Preoperative images of bilateral atypical femoral fractures (AFFs). (a) Bone scintigraphy showing increased uptake in both proximal femurs at follow-up before a left displaced AFF. (b) Radiography showing a left displaced AFF and a right incomplete AFF.

For the inhibition of bone metastasis, zoledronate was initiated at 54 years of age and was continued for 5 years until renal failure. After discontinuation of zoledronate, denosumab was used for 3 years until the detection of AFFs in both proximal femurs on the bone scintigraphy at 62 years of age (Figure 1(a)). Eventually, BMAs had been administered for 8 years. Right hip pain occurred temporarily, whereas left hip pain persisted for a long time. She experienced a left displaced femoral subtrochanteric fracture after falling at the age of 63 years (Figure 1(b)). At that point, the doctors in the previous hospital made a diagnosis of a pathological fracture caused by bone metastasis and consulted with our department for specialized treatment. After the patient was transferred to our hospital, we examined whether that fracture was due to bone metastasis, but no metastatic lesion was noted at the fracture site. In addition, radiography of the fracture area exhibited a beak on the lateral side of the fracture site associated with cortical bone sclerosis, which was characteristic of an AFF (Figure 1(b)) [5]. Considering the long-term administration of BMAs, the fractures were diagnosed as AFFs.

**Figure 2:**
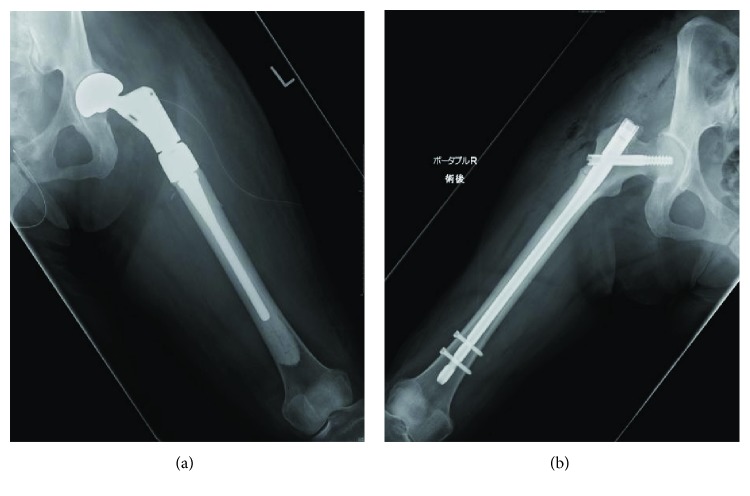
Postoperative images of bilateral atypical femoral fractures (AFFs). (a) Radiography after endoprosthetic reconstruction for the left displaced AFF. (b) Radiography after prophylactic intramedullary nailing for the right incomplete AFF.

For treating the displaced AFF, we could select either IMN or prosthetic reconstruction. For appropriate selection, evaluation of the patient's prognosis is required because recovery of ADL/QOL is the priority in cancer patients with limited life expectancy. The Katagiri score as a predictor of prognosis in patients with skeletal metastasis was high (Table 1(a)) [6], and similarly, the score of another scoring system for metastatic breast cancer was also high (Table 1(b)) [7]. Thus, both predicted a poor prognosis. IMN is a less invasive approach, and it might successfully induce bone healing. However, delayed union/nonunion and implant failure are possible issues. To achieve early weight bearing and avoid these issues, we performed endoprosthetic reconstruction (Zimmer Biomet, OSS Proximal Femur) on the seventh day after the injury (Figure 2(a)). Two weeks after surgery, she achieved walker-assisted gait. After subsequent rehabilitation, prophylactic IMN was performed for the right incomplete AFF (Figure 2(b)). Six months after surgery, the Musculoskeletal Tumor Society (MSTS) score recovered (47%) to almost the preoperative level (50%) before the injury (Table 2). However, she died of breast cancer 1 year and 2 months after the endoprosthetic reconstruction.

## 3. Discussion

BP treatment can reduce the risk of fractures in osteoporosis patients, and this benefit outweighs the disadvantage of AFFs, as the reduction in the occurrence of osteoporotic fractures is much greater than the increase in the risk of AFFs. The incidence of AFFs has been reported to be 0.55 per 1000 BP-treated patients per year [5]. Considering this rarity, some clinicians tend to overlook the importance of AFFs. The incidence of AFFs has been shown to increase around 5 years after the initiation of BPs [8]. Furthermore, changes in the bone advance gradually; hence, BPs are sometimes administered in the long term, and doctors are not aware of the occurrence of AFFs. Once AFFs occur, treatment might be difficult. Incomplete AFFs can be healed with prophylactic IMN at a high rate [3]. Displaced AFFs are more difficult to treat than incomplete AFFs. Displaced AFFs also can be treated successfully with IMN and discontinuation of BMAs; however, some earlier reports have indicated that revision was required in 46% (7/17) of patients owing to nonunion/delayed union [2] and that the mean time to bone union was 11.3 months for displaced AFFs [9]. Another report demonstrated that teriparatide (TPTD) combined with IMN is advantageous, although TPTD is contraindicated in cancer patients with bone metastasis [10]. On the other hand, hip arthroplasty after IMN breakage has been reported to provide good results [11].

Bone metastatic disease often causes intolerable pain and devastatingly impairs ADL in cancer patients. Bone metastasis can be controlled with radiotherapy and/or chemotherapy. Additionally, zoledronate, a third-generation BP, has been frequently used since its beneficial effects on skeletal system-related events were reported [8]. However, the occurrence of AFFs is associated with the long-term use of zoledronate and denosumab. Furthermore, the incidence of AFFs in cancer patients was reported to be higher than that in osteoporosis patients [5]. This previous study mentioned that more attention should be paid to AFFs in cancer patients.

Accurate diagnosis as AFFs is important to avoid overindication of endoprosthesis. Shane et al. stated that pathological fractures are excluded in the diagnostic criteria [12]. In cancer patients, whether pathological fractures with bone metastases are difficult to identify, magnetic resonance imaging (MRI) is very helpful to differentiate AFFs from pathological fractures. In the present case, MRI was conducted and showed no metastatic lesion at the fracture site (data not shown). MRI should be taken before making the decision of the operative method.

In the present report, the patient was predicted to have a poor prognosis, and this information was used to determine the operative method. When a good prognosis is predicted, endoprosthetic reconstruction can be considered excessively invasive. Thus, reliable scoring methods for prognosis prediction are essential for the right decision. In the present study, the scoring systems advocated by Katagiri and Regierer were used, whereas PATHFx program can also provide reliable prediction [13]. With regard to the clinical outcome of endoprosthetic reconstruction for malignant tumors, the implant survival rates at 5 and 10 years were reported to be 84% and 70%, respectively, with the MSTS score of 70.8% [14]. IMN can also provide a good outcome for displaced AFFs, and the primary healing rate has been reported to be 68.7%, and the mean time to union has been reported to be 10.7 months, although there was a significant correlation between malalignment and implant failure/delayed healing time [15]. Therefore, cancer patients with a good prognosis should primarily undergo IMN with care for fracture reduction, and endoprosthetic reconstruction can be a salvage operation after implant failure. In another aspect, IMN seems to have difficulties in treating AFFs with hypertrophy of the lateral cortex associated with bowing deformity [16], which might be a good indication for endoprosthetic reconstruction.

Prognosis is an essential factor in the selection of an operative method, as mentioned above. For bone metastasis, an endoprosthesis should be used in patients with a good prognosis [17], which is in contrast to the approach for AFFs. This difference is associated with the fact that bone metastasis is malignant, whereas AFFs are not tumors. Metastatic lesions can be rarely cured despite chemotherapy and/or radiotherapy. Therefore, complete resection and endoprosthetic reconstruction are preferred when a good prognosis is predicted; otherwise, implant failure can occur at a high rate. On the other hand, displaced AFFs can be successfully treated with IMN if the patient is able to spend the time required for treatment. Thus, IMN should be first considered for patients with a relatively good prognosis. If cancer patients with a poor prognosis undergo IMN, a long period of nonweight bearing is necessary, and patients can experience impairments in ADL/QOL. In the present patient, the postoperative MSTS score recovered to almost the preoperative level, but slightly lower. However, this result does not deny the benefit from endoprosthetic reconstruction. IMN for the present case would provide less satisfactory outcome due to nonweight bearing for a long time. Considering this disadvantage of IMN for displaced AFFs, cancer patients with limited life expectancy need to be able to walk as early as possible after the operation, and endoprosthetic reconstruction is considered a good treatment option in such patients.

## 4. Conclusions

IMN has been generally known as a standard method for displaced AFFs, but delayed union or nonunion is problematic. In cancer patients with poor prognosis who have displaced AFFs, improvement in ADL/QOL is the priority. Endoprosthetic reconstruction for displaced AFFs may be a first-line treatment to acquire early postoperative walking ability for improving ADL/QOL in cancer patients with limited life expectancy.

## Figures and Tables

**Table:** (a) Revised Katagiri scoring system, which is useful for predicting bone metastatic cancers. The parameters are primary site, visceral metastases, laboratory data, ECOG performance status, previous chemotherapy, and multiple skeletal metastases. The prognosis is predicted as follows: 0–3: low risk; 4–6: intermediate risk; 7–10: high risk

Parameter	Value	Points
Primary site	Hormone-dependent breast cancer	0
Visceral metastases	Liver/pleural metastasis	2
Laboratory data	Total bilirubin: 1.65	2
Performance status	PS 4	1
Previous chemotherapy	Yes	1
Multiple skeletal metastases	Yes	1

Total		7

**Table:** (b) An internally and externally validated prognostic score for metastatic breast cancer developed by Dr. Regierer. The parameters are metastasis-free interval, hormone receptor, and metastases of the liver, effusion, brain, bone, bone marrow, soft tissue, and lungs. The score predictions are as follows: 0–8: low risk; 9–14: intermediate; ≥15: high risk

Parameter	Value	Points
Metastasis-free survival	<2 years	3
Hormone receptor	Positive	0
Liver	Yes	7
Effusion	Yes	4
Brain	No	0
Bone	Yes	4
Bone marrow	No	0
Soft tissue	No	0
Lung	No	0

Total		18

**Table 2:** Musculoskeletal Tumor Society (MSTS) scoring for bilateral atypical femoral fracture (AFF) surgery. Preoperative scores before and after the left displaced AFF and postoperative score at 6 months after endoprosthetic reconstruction.

	Preoperation		Postoperation (6 months)
	Before left displaced AFF	After left displaced AFF	
Pain	3: modest/nondisabling	0: severe disabling	5: no pain
Function	1: partial restriction	0: total restriction	1: partial restriction
Emotional	4: intermediate	0: dislikes	3: satisfied
Support	1: one cane	0: impossible to walk	1: one crutch
Walking	3: limited	0: impossible to walk	3: limited
Gait	3: minor cosmetic	0: impossible to walk	1: major cosmetic

Total (%)	15/30 (50%)	0/30 (0%)	14/30 (47%)
